# Characteristics and Management of Community-Acquired Pneumonia in the Era of Global Aging

**DOI:** 10.3390/medsci6020035

**Published:** 2018-04-30

**Authors:** Catia Cillóniz, Diana Rodríguez-Hurtado, Antoni Torres

**Affiliations:** 1 Department of Pneumology, Institut Clinic del Tórax, Hospital Clinic of Barcelona—Institut d'Investigacions Biomèdiques August Pi i Sunyer (IDIBAPS), University of Barcelona—SGR 911—Ciber de Enfermedades Respiratorias (Ciberes), 08036 Barcelona, Spain; 2 Department of Medicine, Full Professor School of Medicine Universidad Peruana Cayetano Heredia, “Hospital Nacional Arzobispo Loayza”, Lima 15082, Perú

**Keywords:** community-acquired pneumonia, elderly, pneumonia, aging

## Abstract

Community-acquired pneumonia (CAP) can occur at any time of life, but its incidence and risk of death are linked to increasing age. CAP in the elderly is a major health problem associated with high rates of readmission, morbidity, and mortality. Since the clinical presentation of pneumonia in the elderly may be atypical, clinicians should suspect pneumonia in older patients presenting symptoms such as falls and altered mental status, fatigue, lethargy, delirium, anorexia, in order to avoid the complications associated with delayed diagnosis and therapy. *Streptococcus pneumoniae* remains the most frequently reported pathogen in this population. However, particular attention should be paid to patients with risk factors for multidrug resistant pathogens, because a large proportion of elderly persons present multimorbidity. Vaccination is one of the most important preventive approaches for CAP in the elderly. In addition, lifestyle-tailored interventions for different modifiable risk factors will help to reduce the risk of pneumonia in elderly persons. Surveillance of etiological pathogens may improve vaccination policies in this population.

## 1. Introduction

The aging population is growing but longevity is not always associated with a positive health status. A large proportion of people ≥65 years old suffer from previous comorbidities, and there is a high prevalence of “multimorbidity” (presence of two or more chronic conditions) that is associated with increased risk of mortality [1,2]. The immunological changes that occur with age called “immunosenescence” (decreased efficiency of the adaptive and innate immune systems) are known to be responsible for the increased susceptibility of elderly persons to infectious diseases and for their limited response to vaccines [3].

**Figure 1:**
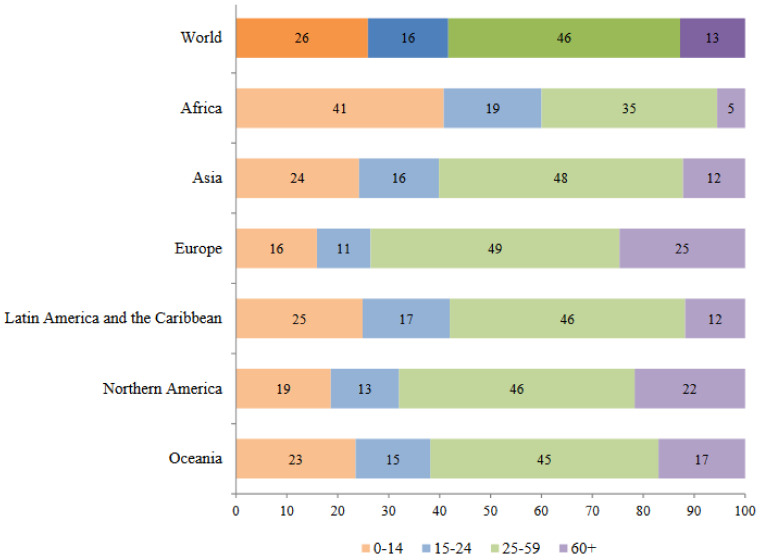
Distribution in percentage by age group and by region. From World Population Prospect: The 2017 Review by United Nations Department of Economic and Social Affairs, United Nations. Reprinted with permission of the United Nations (Copyright 2018).

According to data from the United Nations World Population Prospects, the world population reached 7.6 billion in mid-2017. The population aged 60 or over amounted to 962 million, that is 13% of the total, and this group was growing at a rate of 3% per year. Europe has the highest percentage of people in this age group, at 25% (Figure 1) [4]. 

Community-acquired pneumonia (CAP) is considered the leading infectious cause of sepsis worldwide [5]. It can occur at any time of life, but its incidence and risk of death are linked to increasing age and the presence of comorbidities. Approximately 45% of all CAP cases occur in patients aged 65 or over. In Europe the incidence of CAP in patients ≥65 years old is between 76 and 140 cases per 10,000 adults/year [6,7,8]. In the US, the incidence of CAP in adults between 65 and 79 years old is 63 cases per 10,000 and rises to 164.3 cases per 10,000 adults in the over-80 age group [9]. 

In this study we review the burden of CAP in the elderly population and stress the importance of accurate diagnosis and appropriate treatment. Pneumonia has a disproportionate effect on the elderly, in part because of the age-related changes in the immune system and the high incidence of multimorbidity. 

## 2. Epidemiology

A Spanish study of CAP patients ≥65 years old (2002–2005) reported an overall incidence of 140 cases per 10,000 persons per year: 105 cases per 10,000 for hospitalized CAP and 35 cases per 10,000 for outpatients [7]. Similarly, in a German study [8] that included 388,406 patients (2005–2006), the incidence of CAP reported was 28.5 cases per 10,000 inhabitants per year. The incidence rose to 76.5 cases per 10,000 inhabitants per year in adults aged 60 years or more.

A study in the US carried out between 2010 and 2012 reported an increased incidence of hospitalized CAP with increasing age. The annual incidence of pneumonia in this study was 24.8 cases per 10,000 adults, with the highest rates among adults between 65 and 79 years of age (63.0 cases per 10,000 adults) and in patients ≥80 years old (164.3 cases per 10,000 adults) [9]. More recently, the study by Ramirez et al. [10] (2014–2016) on the epidemiology of CAP reported an overall incidence of 63.4 per 10,000 inhabitants per year. In people aged ≥65 years old, this incidence rose to 209.3 cases per 10,000 inhabitants per year. 

The economic impact of CAP has been addressed in two interesting studies. The first, carried out in the Netherlands, included 195,372 CAP cases and reported that the median costs were dependent on age and type of care, ranging from 344 € (482 €) per episode for 0–9 year old treated as outpatients to 10,284 € (16,374 €) per episode for 50–64 year old admitted to the intensive care unit (ICU). In that study the majority of CAP episodes (64%) occurred among patients aged over 50 years, and these episodes incurred 76% of the costs [11]. The second study from Japan, included 29,619 CAP patients aged ≥65 years and reported median treatment costs of US$ 346 per outpatient CAP episode and US$ 4851 per hospitalized CAP episode [12]. 

**Figure 2:**
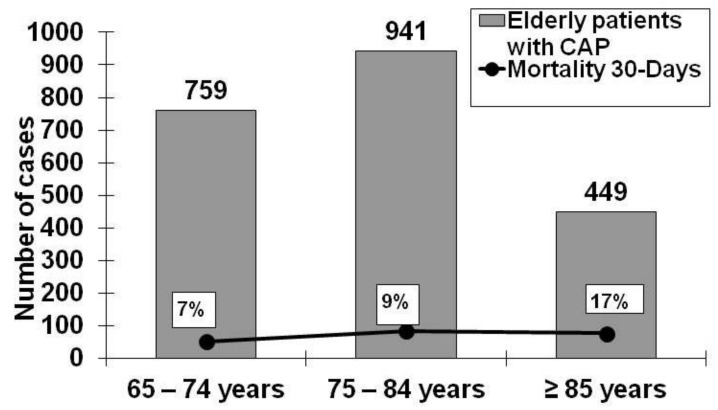
Community Acquired Pneumonia (CAP) by age and percentage of mortality [2].

Mortality in elderly patients with CAP may be 25% higher than in the general population (10%) [2,6,13]. A Spanish study of the impact of age and comorbidities on etiology of CAP which included 2,049 patients, reported that age did not influence the microbial cause itself, but that the mortality rate rose with age (65–74 years old, 6.9%; 75–84 years old, 8.9%; >85 years old, 17.1%; *P* < 0.001) [2] (Figure 2). A study from Argentina of the impact of age and comorbidities on CAP mortality which included 6205 patients reported mortality rates of 8% in patients aged 65–74 years and 14% in patients aged ≥80 years. The authors found that in patients with one comorbidity or less, age 80 or older was a factor that increased mortality [13].

A study from the Netherlands assessed the impact of CAP on health-related quality of life in 562 elderly patients with pneumonia [14]. Interestingly, in immunocompetent patients, hospitalization for suspected pneumonia was associated with a six-fold higher risk of mortality and an average loss of quality of life attributable to pneumonia of 0.13 after one year compared to non-diseased subjects.

## 3. Frailty in the Elderly: A Predisposing Risk Factor for CAP

Frailty may be the result of a variety of diseases and medical conditions. Frail elderly are likely to develop greater dependence and present higher mortality rates when exposed to a stressor [15]. Frailty is age-associated and its prevalence rises steadily: from 4% in the 65–69 year old age group to 7% in the 70–74 year old age group, to 9% in the 75–79 year old age group, to 16% in the 80–84 year old age group and to 26% in the over-85 year old [16,17]. Similar rates were recently reported in a systematic review from Japan where overall frailty among Japanese community-dwelling older people was 7.4% and was more frequent in women (8.1%) than in men (7.6%) [18]. Recently, another systematic review of frailty in Latin America and the Caribbean reported a prevalence in this region of 19.6% [19]. 

The impact of frailty on ICU and 30-day mortality on patients older than 80 years old were reported recently by Flaatten et al. [20] in a study analyzed 5021 patients with a median age of 84 years old, where 311 ICUs from 21 European countries participated. Respiratory and/or circulatory failures were the most frequent causes of ICU admission (41%). Overall ICU and 30-day mortality rates were 22% and 33% respectively. Frailty (measure by the Clinical Frailty Scale) was found in 43% and was independently related to 30-day survival (hazard ratio (HR) 1.54; 95% confidence interval (CI) 1.38-1.73) for frail versus non-frail. The author concluded that in very old (≥80 years old) patients admitted to ICU, the consecutive classes in Clinical Frailty Scale were inversely associated with short-term survival.

All these studies suggest that frailty should be measured in routine clinical practice in order to improve the management of elderly patients with CAP. However, there is no international standard for its measurement. The Frailty Index (FI) proposed by Rockwood et al. [21] is the most frequently used. It includes variables that encompass a range of states, conditions and physiological systems such as mobility, disability, self-rated general health, eyesight, hearing and chronic diseases. 

From a practical point of view, especially in the emergency department, we identify two main groups (as recommended in the Spanish guidelines for the management of elderly patients with CAP) [22]:

(A) Non-frail elderly patients with CAP: patients with no significant comorbidities or mental or social problems and able to perform basic and instrumental daily life activities independently.
(B) Frail elderly patients with CAP: in this group, frailty is further differentiated into mild and moderate-severe.

Elderly patients with mild frailty perform basic activities of daily life independently or almost independently, but within the setting of CAP may present acute functional and/or cognitive impairment. This may increase the grade of comorbidity and dependence for performing instrumental activities of daily life, but these patients are not usually identified as frail [22].

Elderly patients with moderate to severe frailty require help or are dependent for daily life activities and are more likely to present associated severe multimorbidity, polypharmacy, dementia, malnutrition and a situation of social risk [22].

An international multidisciplinary group studying geriatric patients proposed an Integral Geriatric Assessment (IGA) [23] adapted to the Emergency Department (ED) context in order to assess frailty of elderly patients [21,24]. 

Several predisposing factors have been associated with the risk of pneumonia in elderly persons [25,26,27,28,29]. Age by itself is a risk factor for pneumonia because advanced age is associated with a decline in the integrity of physical barriers and protection against invading pathogens, age-related changes in the immune system (immunosenescence), and reduced sensitivity of airway protective cough and swallowing reflexes [25]. 

A case-control study by Jackson et al. [26] including 1173 CAP cases in elderly patients reported that cardiopulmonary disease, low weight and recent weight loss and poor functional status are risk factors for pneumonia in this population. Similarly, the study by Almirall et al. [27] identified oropharyngeal dysphagia as a risk factor for pneumonia in elderly patients. Interestingly, the same authors reported that passive smoking is a risk factor for CAP in patients ≥65 years old who had never smoked [28].

### 3.1. Age-Related Changes (Immunosenescence and Sarcopenia)

The immunosenescence or immunological changes that occur with age involving decreased efficiency of the adaptive and innate immune systems is known to be responsible for the increased susceptibility of the elderly persons to several infectious diseases such as pneumonia [30].

The main consequences of immunosenescence in patients with CAP include the high risk of misdiagnosis of pneumonia because some specific symptoms of lower respiratory tract infection such as cough, chest pain or fever do not reflect the real state of elderly patients with pneumonia [31].

Malnutrition is probably an important factor related to the aging of the immune system. Nutritional supplementation strategies can be improved and reverse some of the effects related to immunosenescence in elderly persons. The supplement provided should include for example zinc, vitamin C, D, E, etc. [32,33,34,35]. 

Sarcopenia is a syndrome featured by age-related loss of skeletal muscle mass, decrease of muscle strength and/or physical performance in the elderly. Due to the sarcopenia is an independent risk factor for adverse outcomes (hospital length stay, readmission, or death) it has become a subject of increased research [36,37,38]. A study from Brazil about the frequency of sarcopenia in elderly patients hospitalized, reported that of the 110 patients included in the study, the frequency of sarcopenia was 22% (representing one in five elderly patients hospitalized), with 10.0% being of the severe type. The factors associated with sarcopenia were age, clinical profile in admission and smoking [39]. Improving muscle strength and physical performance may be improved with exercise interventions. Also, the nutritional intervention plays an important role in persons with sarcopenia [40,41]. 

## 4. Clinical Presentation of Pneumonia

Elderly persons suffer from a variety of comorbidities that affect the integrity of their natural resistance to infections such as pneumonia and increase the risk of morbidity and mortality [3]. In a Spanish study [2] of the impact of age and comorbidities on the etiology of CAP, 80% of the cases presented at least one comorbidity (chronic respiratory disease, diabetes mellitus, chronic cardiovascular disease, neurological disease, chronic liver disease, and chronic renal disease) at the following rates according to age group: 65–74 years old, 77.6%; 75–84 years old, 80.6% and ≥85 years old, 80.8%. In this study the most frequent comorbidity was chronic pulmonary disease, presented by 54.1% of the cases.

Immunosenescence is responsible for the reduced response to infection and the increase in pathological disorders in the elderly population [3]. It increases the risk of misdiagnosis of pneumonia or delayed diagnosis because certain specific symptoms of infection such as cough, fever, chills, and chest pains do not reflect the real state of the elderly with pneumonia [42].

The main symptoms associated with pneumonia in elderly persons are falls, altered mental status (i.e., delirium), fatigue, lethargy, delirium, anorexia, tachypnea, tachycardia, and, less commonly, pleuritic pain, cough, fever, and leukocytosis (Table 1) [43,44]. In 2014 an interesting study was published on the association of the presence of delirium in elderly patients with CAP and in-hospital mortality [45]. In this study Pieralli et al. [45] included 443 patients with a mean age of 81.8 years old; 31% presented more than three comorbidities and the in-hospital mortality rate was 12%. Risk factors related to in-hospital mortality in this study were: chronic obstructive pulmonary disease (COPD) (odds ratio (OR) 6.21), occurrence of at least one episode of delirium (OR 5.69), male sex (OR 5.10), and CURB-65 (confusion, urea, respiratory rate and age 65 years old) score (OR 3.98).

Related to the study mentioned above, a Canadian study [46] of the impact of multimorbidity on the clinical outcome of CAP found that 2240 of the 5565 patients included (40%) were ≥65 years old and that 71% of these patients presented multimorbidity defined as presenting as at least two previous comorbidities. The authors concluded that the presence of multimorbidity was independently associated with death, hospitalization or return to emergency department within 90 days of discharge. The data highlighted the importance of considering previous comorbidities in elderly patients in order to prevent worse outcomes. 

It is also important to remember that pneumonia sometimes presents as an exacerbation or decompensation of previous comorbidities (diabetes mellitus, cardiac disease, chronic pulmonary disease) and that in approximately 30% of the cases the radiographic findings are inconclusive or difficult to interpret [22]. 

Elderly patients have an inadequate inflammatory response to infection because of immunosenescence [47,48], which can lead to an underestimation of the severity of the pneumonia. Many biomarkers of infection, such as leukocyte count, C-reactive protein (CRP), and procalcitonin have been found to play a role in the early diagnosis and prognosis of pneumonia, especially CAP. But the data on biomarkers of infections in elderly patients with CAP are limited [49]. 

## 5. Evaluation of Severity

CAP in elderly patients is often more severe than in younger patients and may result in poor prognosis. Several predictors of mortality in elderly patients with CAP have been reported:

The prognostic value of glucose levels in elderly patients with pneumonia was investigated in the study by Akirov et al. [50] in a cohort of 2164 elderly patients. These authors found that in elderly non-diabetic patients hospitalized for pneumonia, moderate and markedly elevated blood glucose levels on admission were associated with increased short-term and long-term mortality.

The value of the neutrophil-to-lymphocyte ratio (NLR) was evaluated by Cataudella et al. [51] in a cohort of 196 patients with CAP. The authors compared NLR with the Pneumonia Severity Index (PSI) and CURB-65 [52] severity scores, and with infection markers CRP and white blood cell count (WBC). The authors reported that NLR predicted 30-day mortality (*p* < 0.001) and performed better than PSI (*p* < 0.05), CURB-65, C-reactive protein, and WBC (*p* < 0.001) for predicting prognosis in elderly patients with CAP. These data corroborate the results of the 2009 study by Thiem et al. [53] which included 391 elderly patients with CAP; they found no association between CRP or WBC and mortality, but severity scores PSI and CURB-65 were significantly associated with mortality and treatment in the ICU.

Two studies addressed the impact of previous comorbidities on the prognosis of pneumonia. The first study from Canada [54] investigated the predictive factors of in-hospital mortality and readmission in 717 elderly patients with CAP. The authors found that chronic comorbidities were the main predictors of mortality and readmission in elderly patients with pneumonia. Interestingly, vitamin E supplementation was identified as a protective factor. The second study, from Argentina [55], investigated the impact of age and comorbidities on mortality in CAP patients according to age group (<65, 65–79 and ≥80 years old). The authors found an association between the presence of previous comorbidities and poor outcome in CAP patients. The study also reported that age ≥80 years old is a factor for increased mortality when patients presented one comorbidity or less. 

The PSI [56] and the CURB-65 [52] devised specifically to allow more objective decision-making regarding hospitalization, are the two most commonly used scores for predicting short-term mortality in CAP. However, a major limitation of the PSI score when applied in elderly patients is its heavy weighting by age and comorbidity; these scores also have low sensitivity and specificity for predicting ICU admission and do not consider important factors such as psychosocial variables, infrequent comorbidities, or patient preferences regarding treatment. The limitations of the CURB-65 score include the fact that it does not include data such as hypoxemia, electrolyte disturbance or the inability to take oral medications which might influence the severity of CAP, especially in elderly patients. 

The study by Chen et al. [57] of the performance of the PSI and CURB-65 scores in young (18–64 years old), elderly (65–84 years old) and very old (≥85 years old) populations found poorer performance of both scores (especially PSI) in the elderly and very old groups. This may be due to an excessive weight given to the age variable. The author proposed a modified score that excluded age in this population. 

Recently, a Spanish study [58] investigated the predictive value of a combined model incorporating the Barthel Index (BI) and PSI in patients aged ≥65 years for predicting mortality in elderly patients with CAP. The authors included 1919 patients ≥65 years old with CAP. Sixty-one per cent had severe CAP with PSI IV-V and 40% had BI ≤90 points. The authors demonstrated that the combination of BI ≤90 points and PSI IV-V was the greatest risk factor for mortality in the elderly population with CAP. 

This study highlights the importance of paying attention to specific factors that may predict a poor outcome in this population, such as previous bed confinement, abnormal mental status, absence of chills, or nutritional status. Similarly, a recent study by Pieralli et al. [59] addressed the role of performance status evaluated by the Eastern Cooperative Oncology Group (ECOG) score in predicting 30-day mortality in patients hospitalized for CAP. The ECOG score (graded from zero to five points) was used by the oncologist to assess functional status and treatment strategies and in order to predict outcome in elderly people hospitalized for pneumonia [60]. The authors performed a two-year prospective study including 216 CAP patients, more than 76% of whom were ≥70 years old, with a balanced proportion of males/females. Thirty-two per cent presented severe CAP according to CURB-65 score (2–3) and 28% had severe disability according to their ECOG (3–4). Thirty-day mortality was 15%. In a multivariate analysis, progression in ECOG score independently increased the probability of 30-day mortality (HR 2.19), and an ECOG score of three or four was associated with a four-fold increase in 30-day mortality (HR 4.07). 

In patients classified at low risk of mortality by the CURB-65 score (0-2), and ECOG score three or four is highly predictive of mortality. The authors demonstrated that functional status is directly related to outcome in elderly patients hospitalized for CAP. Physicians should take account this very simple and fast score that might help stratify patients at risk of short-term mortality in CAP, especially in patients classified at low risk by CURB-65 score.

Rates of ICU admission for elderly patients are increasing [60,61,62]. However, intensive care may not benefit those with irreversible disease or extreme frailty [63]. SMART-COP [61] and the criteria proposed by the American Thoracic Society/Infectious Disease Society of America (ATS/IDSA) guidelines [5] are the most commonly used score for predicting admission to the ICU. However, the absence of age in the minor and major ATS/IDSA criteria and the fact that the SMART-COP sets, the cutoff for age at 50 years old, limit the use of these scores in the elderly population. 

The study by Al-Dorzi et al. [62], which included 9493 ICU patients, aimed to compare the clinical characteristics, ICU management and clinical outcomes of elderly patients aged ≥80 years and younger patients (50–64.9 years old and 65–79.9 years old). The authors found that patients ≥80 years old represented 8% of all ICU admissions. The most frequent comorbidities in this group of patients were chronic cardiac disease (32%) and respiratory disease (22%), and the most frequent causes of ICU admission were cardiovascular (30.9%) and respiratory (40.4%) conditions. Thirty-three per cent of the patients presented with sepsis and 77% needed mechanical ventilation. The authors also reported that hospital mortality increased gradually with age and was highest (54.6%) in patients over the age of 80. 

## 6. Therapy

Several studies have reported a direct association between early appropriate antibiotic therapy and improved clinical outcomes, especially in the elderly population [63,64,65]. A Spanish study [2] analyzing the microbial etiology in a cohort of 2149 CAP patients divided by age (65–74 years old, 75–84 years old and ≥85 years old) reported that microbiological diagnosis in CAP decreases steadily with age: 65–74 years old, 44%; 75–84 years old, 41%; and ≥85 years old, 31% (*p* < 0.001). For this reason, empirical antibiotic therapy is mandatory and is based on the severity of pneumonia infection, the risk factors for multidrug-resistant pathogens, and the local epidemiology.

Current international guidelines for the management of CAP do not provide specific recommendations for elderly patients [5,66,67]. However, some studies have reported that adherence to guidelines in the treatment of elderly patients with CAP is associated with better outcomes. In the study by Arnold et al. [68] of clinical outcomes in elderly patients (≥65 years old) with CAP, 975 patients adhered to the 2007 ATS/IDAS guidelines whereas 660 did not. Adherence to guidelines was associated with shorter time to clinical stability, shorter hospital stay, and lower in-hospital mortality.

In addition, although there are few studies that evaluate the cost effectiveness of adherence to antibiotic guidelines in CAP. An international, multicenter observational study for patients age ≥65 years or older hospitalized with CAP from 2001 to 2007 addressed by Egger et al. [69] reported that adherence to the 2007 IDSA/ATS guidelines regarding empiric antibiotic therapy for elderly patients hospitalized with CAP was cost effective compared to non-adherence in non-ICU patients, but was not the most cost effective strategy in ICU patients [69,70]. 

For antibiotic therapy in elderly patients, it is important to take account of age-related changes that modify tolerability, metabolism, and excretion and the fact that multimorbidity, frailty and polypharmacy add to the risk of drug-drug interactions (Table 2). Also, the higher risk of multidrug resistant pathogens in elderly patients is an important issue when evaluating the initiation of antibiotic therapy in these patients. 

Several studies on CAP in different parts of the world have reported that *Streptococcus pneumoniae* (pneumococcus) remains the leading cause of pneumonia in the elderly population [2,71,72,73,74]. In the study by Cilloniz et al. [2] of the impact of age on the microbial etiology of CAP, pneumococcus was the most frequently reported microorganism in all age groups, as follows: 41% in the 65–74 years old group, 39% in the 75–84 years old group, and 49% in the >85 years old group. CAP cases caused by two or more pathogens (polymicrobial etiology) were the second most frequent and were reported as follows: in 16% in the 65–74 years old group, 13% in the 75–84 years old group, and 11% in the >85 years old group. Intracellular pathogens (*Legionella pneumophila*, *Mycoplasma pneumonia*, *Coxiella burnetii* and *Chlamydophila pneumoniae*) were the third cause of pneumonia and were reported as follows: 16% in the 65–74 years old group, 13% in the 75–84 years old group, and 10% in the >85 years old group. Respiratory viruses were the fourth most frequent cause of CAP 8% in the 65–74 years old group, 15% in the 75–84 years old group, and 11% in the >85 years old group. *Staphylococcus aureus*, Enterobacteriaceae, *Pseudomonas aeruginosa* and *Haemophilus influenzae* were also more frequent in patients with at least one comorbidity.

The differences in microbial etiology between patients in nursing-homes and those in the community remain controversial. Two European studies on nursing home patients with CAP found the etiology to be similar to that reported in CAP patients [71,74]. However, clinicians should carefully consider the presence of specific risk factors associated with multidrug-resistant pathogens such as prior antibiotic therapy, chronic pulmonary disease, admission to a nursing home and prior hospitalization [72,75,76,77] (Table 3).

Another important aspect regarding the cause of pneumonia in the elderly is the frequency of aspiration in this population [78]. The risk factors associated with aspiration are neurologic impairment (i.e., stroke, altered mental status, decreased consciousness, seizures or drug overdose), lung disease, diabetes mellitus, malnutrition, proton pump inhibitor use, antipsychotic drug use or sedatives, endotracheal intubation and bronchoscopy [79,80]. 

International Guidelines and Recommendations for Antimicrobial Treatment of CAP of the British Toracic Society (BTS), ATS/IDSA and European Respiratory Society/ European Society of Clinical Microbiology and Infectious Diseases (ERS/ESCMID) are summarized in Table 4, Table 5 and Table 6.

## 7. Prevention

International guidelines [57,67,68] recommend specific measures for preventing pneumonia. The most important of these is the use of pneumococcal vaccines (polysaccharide and conjugate) and influenza vaccines in all older adults and in younger persons with medical conditions that place them at a high risk of pneumonia morbidity and mortality. In addition, the lifestyle interventions for modifiable risk factors for CAP will help to reduce the risk of pneumonia in elderly persons [29,81]. 

### 7.1 Influenza Vaccine 

Influenza affects people of all range of age. However, severe illness and complications are more frequent in people who are most vulnerable such as elderly persons, and persons with chronic medical conditions such as diabetes, cardiovascular disease, respiratory disease or immunosuppresion. During winter seasons in Europe, influenza epidemics are associated with high morbidity and mortality according the European Centre for Diseases Prevention and Control (ECDC) reports. The study by Vestergaard et al. [82] which included data from 19 European countries or regions that investigated the estimated cumulated influenza-attributable mortality in the 2016/17 winter season, reported 137 (range: 76–302) deaths per 100,000 population in the 2016/17 winter season (until week 8/2017), the authors explain that the circulation of influenza virus A(H3N2) and the cold weather snaps contributed in some countries to the excess mortality in elderly people. The pattern was similar to the last major influenza A(H3N2) season in 2014/15 in Europe.

The recent review by Demicheli et al. [83] about the effects (efficacy, effectiveness and harm) of vaccines against influenza in the elderly. The authors analyzed results of vaccine efficacy against influenza of eight Randomized controlled trials. The authors concluded that elderly people receiving the influenza vaccine may have a lower risk of influenza (from 6% to 2.4%), and probably have a lower risk of influenza-like illness compared with those who do not receive a vaccination over the course of a single influenza season (from 6% to 3.5%). 

Influenza vaccine is based on the virus circulated in the previous season and made available in advance on the influenza season. The recommended composition of viruses in the flu vaccine is defined annually by the WHO.

### 7.2 Pneumococcal Vaccine

A recent published study reported a notable burden of pneumococcal CAP in European adults, particularly among the elderly [84]. Currently two types of vaccine are available: polyvalent pneumococcal polysaccharide vaccine (PPV) and the pneumococcal conjugate vaccines (PCV). PPV contains amounts of unconjugated purified capsular polysaccharides of each of the 23 pneumococcal serotypes included, and in PCV, the capsular polysaccharides are conjugated to a carrier protein to enhance immunogenicity [85].

In 2015 was published the results of a randomized double-blind, placebo-controlled study (CAPiTA) conducted in the Netherlands, involving 84,496 adults aged 65 years and over (2008–2013) that evaluate the efficacy of PPV13 vaccine in preventing the first episode of vaccine-type strains of pneumococcal CAP, non-bacteremic and noninvasive pneumococcal CAP, and invasive pneumococcal disease (IPD). The results showed 45.5% (95.2% CI, 21.8-62.5; *p* < 0.001) efficacy of PCV13 gains all vaccine type pneumococcal CAP, 45% (95.2% CI, 14.2–65.3; *p* < 0.001) efficacy against vaccine-type non-bacteremic pneumococcal CAP and 75% (95.2% CI, 41.4–90.8; *p* < 0.001) efficacy against vaccine type IPD among adults aged ≥65 years [86]. These published results led to the licensing in many countries of conjugated vaccine for elderly persons.

#### 7.2.1. Current Advisory Committee on Immunization Practices (ACIP) Recommendations for PCV13 and PPV23 in Adults [87,88]

Pneumococcal vaccine in naïve persons ≥65 years old: vaccination naïve persons should receive a single dose of PCV13 first, followed by a dose of PPV23 ≥1 year later.

Prior vaccination with PPV23 at age ≥65 years: adults aged ≥65 years who have previously received ≥1 doses of PPV23 should also receive a dose of PCV13 if they have not yet received one. A dose of PCV13 should be given ≥1 year after receipt of the most recent PPV23 dose. In the case of patients who need to repeat PPV23, the period between administration of PCV13 and the new dose of PPV23 should be ≥1 year, and five years since the most recent dose of PPV23.

#### 7.2.2. Current ACIP Recommendations for PCV13 and PPV23 in Patients Aged 19–64 Years with Immunocompromising Conditions or Anatomical or Functional Asplenia, Cerebrospinal Fluid Leaks or Cochlear Implants

- Pneumococcal vaccine-naïve: initial dose of PCV13 followed by one dose of PPV23 (≥8 weeks following dose of PCV13). A second dose of PPV23 is recommended five years after the first PPV23 dose for persons aged 19–64 with immunocompromising conditions or with anatomical or functional asplenia.
- Previously vaccinated with PPV23: one dose of PCV13 (≥1 year after receipt of most recent PPSV23 dose) followed by one dose of PPV23 (≥8 weeks following dose of PCV13 and ≥5 years since most recent dose of PPV23).

### 7.3 Other Preventive Measures:

In 2013, Torres et al. [89] investigated the association between the incidence of CAP and group of age, chronic comorbidities and lifestyle factors in adult population from Western Europe. Different modifiable risk factors related to lifestyle were reported in this study and gave the opportunity to intervene on them. Such modifiable risk factors were:

- Higher consumption of alcohol (reduce alcohol consumption).
- Exposure to cigarette smoke (smoking cessation).
- Dental hygiene (improve oral hygiene and annual visits to the dentist).
- Being underweight (ensure good nutritional status).
- Contact with children (avoid contact with children with lower respiratory infections).

## 8. Conclusions

Community-Acquired Pneumonia is a common cause of hospitalization in the elderly and represents a serious health problem all over the world. Expert teams combining pulmonary specialists and infectious diseases and geriatric specialists are needed for its management. Clinicians should suspect pneumonia in older persons with atypical presentations in order to avoid the complications associated with delayed treatment. 

It is important to identify elderly patients with a risk factor for multidrug resistant pathogens in order to ensure adequate treatment. Establishing functional status in elderly patients with CAP is also essential since it is related to clinical outcomes and treatment response.

## Figures and Tables

**Table 1:** Clinical Features of Community Acquired Pneumonia in the Elderly.

Signs and Symptoms
Delirium/acute confusional status
Decreased appetite
Urinary incontinence
Pleuritic pain
Leukocytosis
Shortness of breath
Fever
Cough

**Table 2:** Risk factors for specific microorganisms in Community-Acquired Pneumonia [75,76,77].

Microorganism	Risk Factor
*Streptococcus pneumoniae*	Dementia, seizure disorders, congestive heart failure, cerebrovascular disease, chronic obstructive pulmonary disease, HIV infection, black race, overcrowded living conditions, and smoking
*S. pneumoniae* resistant to ß- lactam	Use of ß- lactam (prior 3-6 months), Prior hospitalization (3 months), Aspiration, Previous episodes of pneumonia in the last year, Non-invasive disease, >65 years old Attendance in day care, COPD
*S. pneumoniae* resistant to macrolide	Recent macrolide use (prior 1-3 months), >65 years old, Attendance in day care centers, Recent hospitalization
*S. pneumoniae* resistant to fluoroquinolones	Prior exposition to fluoroquinolones, Nursing home, Nosocomial infection, Penicillin resistance, COPD
*Staphylococcus aureus*	Advanced age, underlying lung disease, and previous antibiotic use
Methicillin-resistant *Staphyloccus aureus* (MRSA)	Previous MRSA infection or colonization, residence in a nursing home or extended-care facility, cerebrovascular disease, diabetes, chronic renal failure, hospitalization for ≥2 days in the preceding 90 days, prior intravenous antibiotic therapy within the last 30 days
Community-acquired Methicillin-resistant *Staphyloccus aureus* *(CA-MRSA)*	History of viral upper respiratory infection, smoking, recent hospitalization, and chronic pulmonary disease
*Haemophilus influenzae*	Chronic obstructive pulmonary disease treated with antibiotics or oral steroids within the previous 3 months
*Pseudomonas aeruginosa*	Pulmonary comorbidity is the major risk factor
Multidrug-resistant *Pseudomonas aeruginosa*	Prior antibiotic treatment (1 month)

**Table 3:** Microbial etiology of Community-Acquired Pneumonia in the elderly patients [2,9,69,70,72,76].

Microorganism	Frequency
*Streptococcus pneumoniae*	10%–50%
Intracellular bacteria	2%–15%
*Haemophilus influenzae*	1%–10%
Respiratory viruses	2%–20%
*Pseudomonas aeruginosa*	1%–15%
*Staphylococcus aureus*	1%–7%
Polymicrobial etiology	2%–13%
*Enterobacteriaceae*	1%–3%
Aspiration	10%

**Table 4:** Guidelines for the Management and Treatment of Community-Acquired Pneumonia.

**BTS Guidelines** [64]
**Outpatients/Low Severity** CURB-65 score of 0 to 1. Empirical therapy is primarily directed at *S. pneumoniae*. Treat with oral amoxicillin (preferred agent, dose of 500 mg, three times daily), or doxycycline, or clarithromycin for patients hypersensitive to penicillin.
**Inpatients Moderate Severity** CURB-65 score of 2.Treat with oral amoxicillin plus clarithromycin. When oral therapy is contraindicated, the preferred parenteral choices include intravenous amoxicillin or benzylpenicillin, together with clarithromycin. Alternative for patients intolerant to penicillins or macrolides: doxycycline, moxifloxacin, or levofloxacin. IV recommendations include levofloxacin monotherapy, a second generation (e.g., cefuroxime), or a third-generation (e.g., cefotaxime or ceftriaxone) cephalosporin together with clarithromycin.
**Inpatients High Severity** CURB-65 score of 3 to 5. Treat immediately after diagnosis. Treat with an intravenous combination of a broad-spectrum β-lactamase stable antibiotic such as co-amoxiclav, together with a macrolide, such as clarithromycin is preferred. In patients allergic to penicillin, a second-generation (e.g., cefuroxime) or third-generation (e.g., cefotaxime or ceftriaxone) cephalosporin can be used instead of co-amoxiclav, together with clarithromycin. Patients with *Pseudomonas* infection: ceftazidime plus gentamicin or tobramycin (dose monitored). Alternatively, ciprofloxacin or piperacillin, plus gentamicin or tobramycin (dose monitored).

BTS: British Toracic Society; CURB-65: confusion, urea, respiratory rate and age 65 years old.

**Table 5:** ATS/IDSA Guidelines for the Management and Treatment of Community-Acquired Pneumonia.

ATS/IDSA Guidelines [5]
**Outpatients/low severity:** PSI OR CURB-65 score to guide outpatient treatment. Treat previously healthy patients with low risk of drug-resistant pneumococci with a macrolide (azithromycin, clarithromycin, or erythromycin) (strong recommendation; level I evidence) or doxycycline (weak recommendation; level III evidence). Treat patients with a high risk of drug-resistant pneumococci with a fluoroquinolone or β-lactam plus macrolide. Presence of comorbidities, use of immunosuppressing drugs, use of antimicrobials within the previous three months, or other risks of DRSP infection: Treat with respiratory fluoroquinolone (moxifloxacin or levofloxacin [750 mg]) (strong recommendation; level I evidence). β-lactam plus a macrolide (high-dose amoxicillin [e.g., 1 g, three times daily] or amoxicillin-clavulanate [2 g, twice daily] is preferred; alternatives include ceftriaxone, cefpodoxime, and cefuroxime [500 mg, twice daily]; doxycycline [level II evidence] is an alternative to the macrolide) (strong recommendation; level I evidence). In regions with a high rate (>25%) of infection with high-level (MIC, ≥16 mg/mL) macrolide-resistant *Streptococcus pneumoniae*: Consider the use of alternative agents listed above for any patient, including those without comorbidities (moderate recommendation; level III evidence).
**Patients directly admitted to hospital:** Treat with a respiratory fluoroquinolone (strong recommendation; level I evidence) or a β-lactam plus a macrolide (strong recommendation; level I evidence). Preferred β-lactam agents include cefotaxime, ceftriaxone, and ampicillin; ertapenem for selected patients; with doxycycline [level III evidence] as an alternative to the macrolide. A respiratory fluoroquinolone should be used for penicillin-allergic patients.
**Patients who require ICU admission:** Treat with β-lactam (e.g., cefotaxime, ceftriaxone, or ampicillin-sulbactam) plus either azithromycin (level II evidence) or a fluoroquinolone (level I evidence) (strong recommendation). Alternatively, a respiratory fluoroquinolone and aztreonam are recommended for penicillin-allergic patients.
**Patients with *Pseudomonas* infection:** Treat with either an antipneumococcal, antipseudomonal β-lactam (e.g., piperacillin-tazobactam, cefepime, imipenem, or meropenem) plus either ciprofloxacin or levofloxacin (750 mg dose are recommended), or the above β-lactam plus an aminoglycoside and azithromycin, or the above β-lactam plus an aminoglycoside and an antipneumococcal fluoroquinolone. For patients allergic to penicillin the above β-lactam should be substituted with aztreonam (moderate recommendation; level III evidence). Patients with community-acquired methicillin-resistant *Staphylococcus aureus* (CA-MRSA) infection: Add vancomycin or linezolid to standard CAP therapy (moderate recommendation; level III evidence).

ATS/IDSA: American Thoracic Society/Infectious Disease Society of America; PSI: pneumonia severity index; DRSP: Drug-resistant *Streptococcus pneumoniae*; ICU: intensive care unit.

**Table 6:** ERS/ESCMID Guidelines for the Management and Treatment of Community-Acquired Pneumonia [65].

Low Severity	Moderate—High Severity
CURB-65 to guide outpatient treatment Treatment: * Aminopenicillin ± macrolide * Aminopenicillin/ß-lactamase inhibitora ± macrolide * Non-antipseudomonal cephalosporin II or III + macrolide * Cefotaxime or ceftriaxone ± macrolide * Penicillin G ± macrolide	ICU admission: acute respiratory failure, severe sepsis or septic shock and radiographic extension of infiltrates/ severely decompensated comorbidities No risk factors for *Pseudomonas aeruginosa:* Non-antipseudomonal cephalosporin III + macrolide or non-antipseudomonal cephalosporin III + moxifloxacin or levofloxacin Risk factors for *P. aeruginosa:* Antipseudomonal cephalosporin or acyl-ureido-penicillin/ß-lactamase inhibitor or carbapenem (meropenem preferred) plus ciprofloxacin or plus macrolide + aminoglycoside (gentamicin, tobramycin or amikacin)

ERS/ESCMID: European Respiratory Society/ European Society of Clinical Microbiology and Infectious Diseases.
